# Accelerating the science and practice of psychology beyond WEIRD biases: Enriching the landscape through Asian psychology

**DOI:** 10.3389/fpsyg.2022.1054519

**Published:** 2022-12-22

**Authors:** Paul T. P. Wong, Richard G. Cowden

**Affiliations:** ^1^Trent University, Peterborough, ON, Canada; ^2^Human Flourishing Program, Harvard University, Cambridge, MA, United States

**Keywords:** Asian psychology, global psychology, indigenous psychology, multiculturalism, WEIRD psychology, psychological science

## Abstract

More than a decade has passed since major concerns emerged about the WEIRD-centric focus of mainstream psychological science. Since then, many calls have been made for the discipline of psychology (and other disciplines within the social sciences) to become more broadly representative of the human species. However, recent evidence suggests that progress toward improving the inclusivity and generalizability of psychological science has been slow, and that the dominance of WEIRD psychology has persisted. To build a more comprehensive psychological science that truly represents the global population, we need strategies that can facilitate more rapid expansion of empirical evidence in psychology beyond WEIRD biases. In this paper, we draw on several examples (i.e., non-duality and dialectical interaction, Wu-Wei, Zhong Yong) to illustrate how principles of Asian psychology could contribute to reshaping mainstream psychology. We discuss some strategies for advancing a global psychological science, along with some complementary practical suggestions that could enrich the WEIRD-centric landscape of current psychological science.

## Introduction

More than a decade ago, Arnett (2008) published a seminal paper suggesting that up to 95% of participants included in publications within premier American Psychological Association (APA) journals during the last 20 years were from populations that represented approximately 12% of the global population (68% of samples were from the United States). This finding ignited concerns that psychological science (and social science more generally) might be disproportionately skewed toward and narrowly representative of people from societies that are Western, Educated, Industrialized, Rich, and Democratic (WEIRD), and ushered in calls to build a more inclusive body of social scientific research that is representative of all humanity (Henrich et al., 2010).

Almost 15 years later, Thalmayer et al. (2021) evaluated progress toward greater diversification of psychological science beyond WEIRD contexts by repeating Arnett’s (2008) analysis for the years 2014–2018. They found that up to 93% of participants in the publications during the study period were from WEIRD societies (62% of samples were from the United States). Although Thalmayer et al.’s (2021) analysis focused on top-tier psychology journals published by the APA (and therefore may not be entirely representative of the broader psychological literature), other similar reviews in psychology have reported comparable findings (e.g., Hendriks et al., 2019). Hence, it appears that the WEIRD-centric emphasis of psychological science has largely persisted.

Tremendous contributions have been made by WEIRD-centric psychology toward understanding and promoting human flourishing. However, the WEIRD-centricity that characterizes mainstream psychology presents an opportunity to develop strategies aimed at building a more inclusive and representative psychological science (Lomas, 2018). Without such transformation, meta-analytic evidence, theory development, and policymaking decisions are more likely to be informed by WEIRD psychosocial functioning and biased toward people living in WEIRD societies. In this paper, we draw on several examples to illustrate how principles of Asian psychology might contribute to enriching mainstream psychology. Against this backdrop, we offer some strategies for advancing and accelerating change toward a more culturally responsive and inclusive global psychology (Hwang, 2015, 2019), and provide some complementary practical suggestions that might help reshape the WEIRD-centric landscape of current psychological science.

## Non-duality and dialectical interaction

An important contribution of Asian psychology is the principle of non-duality, which emphasizes a continuous process of dialectical interaction between opposites that form a unified whole. The principle of non-duality implies that any attempt to conceptualize or measure a psychological phenomenon ought to recognize the reality of human existence as involving dialectical interplays between opposing dimensions. One example of this is the bifocal approach to locus of control, which does not consider internal and external locus of control as opposite poles on a single dimension, but rather as two independent and interactive dimensions. Only this conception can explain why Chinese students might score high on measures of both internal (e.g., attributing success to ability and effort) and external (e.g., attributing success to parental support, good teachers, and good luck) locus of control (Wong and Sproule, 1984). Findings along similar lines have emerged in other studies, such as school-aged children who report both high mathematics anxiety and mathematics motivation (Wang et al., 2018) or young adult athletes who endorse both approach and avoidance achievement goals (Cowden et al., 2021a).

**Figure 1:**
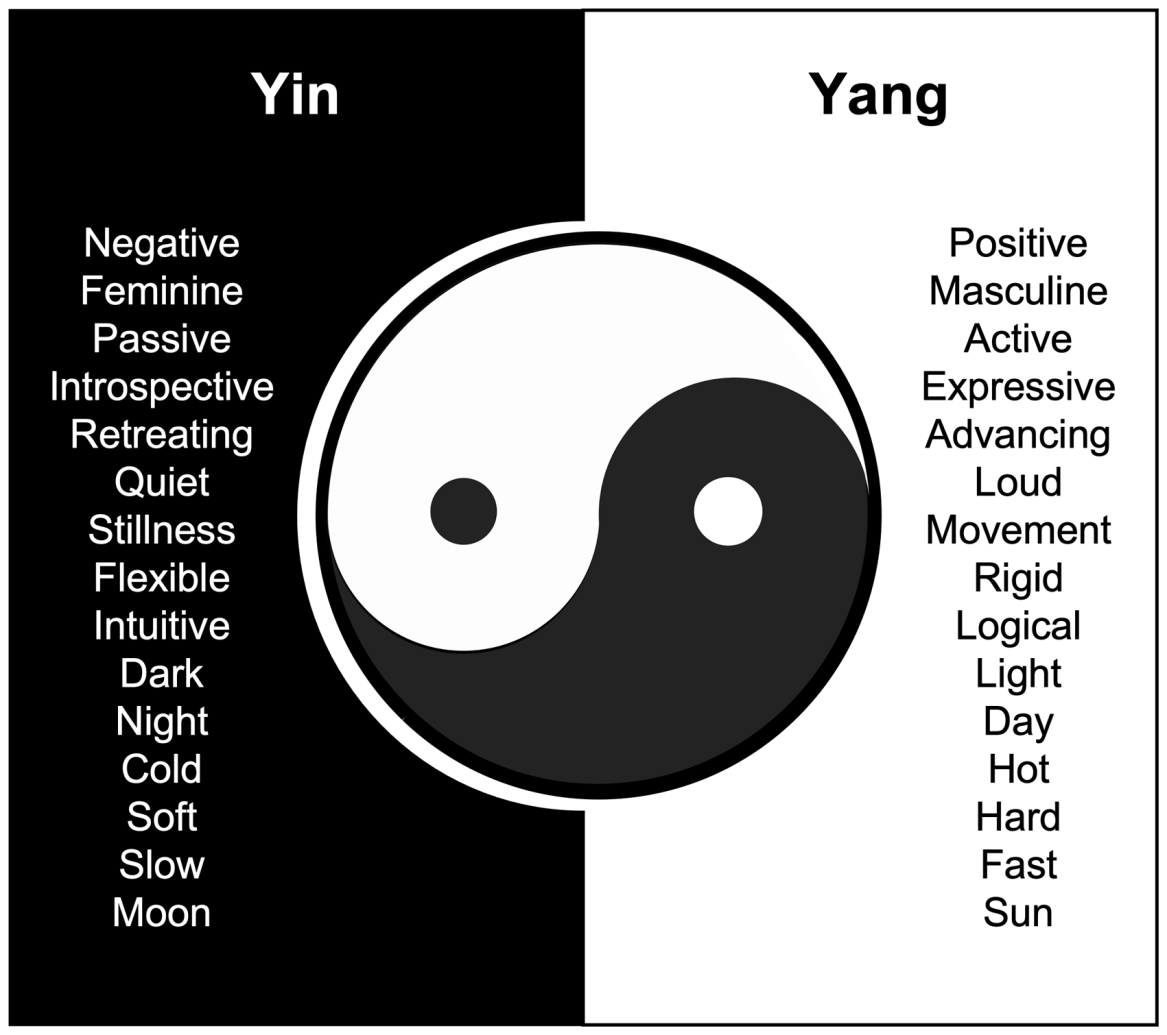
Yin-Yang symbol and dialectics.

Non-duality looks at everything in terms of wholeness based on Yin-Yang integration of independent but interactive opposing dimensions (see Figure 1). A key advantage of this approach is that it enables participants to express their mental or emotional states anywhere in a two-dimensional space. This non-duality perspective can also encourage individuals to hold two opposing ideas, emotions, or values in both hands and find a way to live with this constant tension. Not only can this Asian worldview change how we typically study the mind and human behavior, but it has clinical utility in helping people become aware of Yin-Yang dialectics and figure out more adaptive ways to embrace and transcend opposite forces (Wong, 2016).

**Figure 2:**
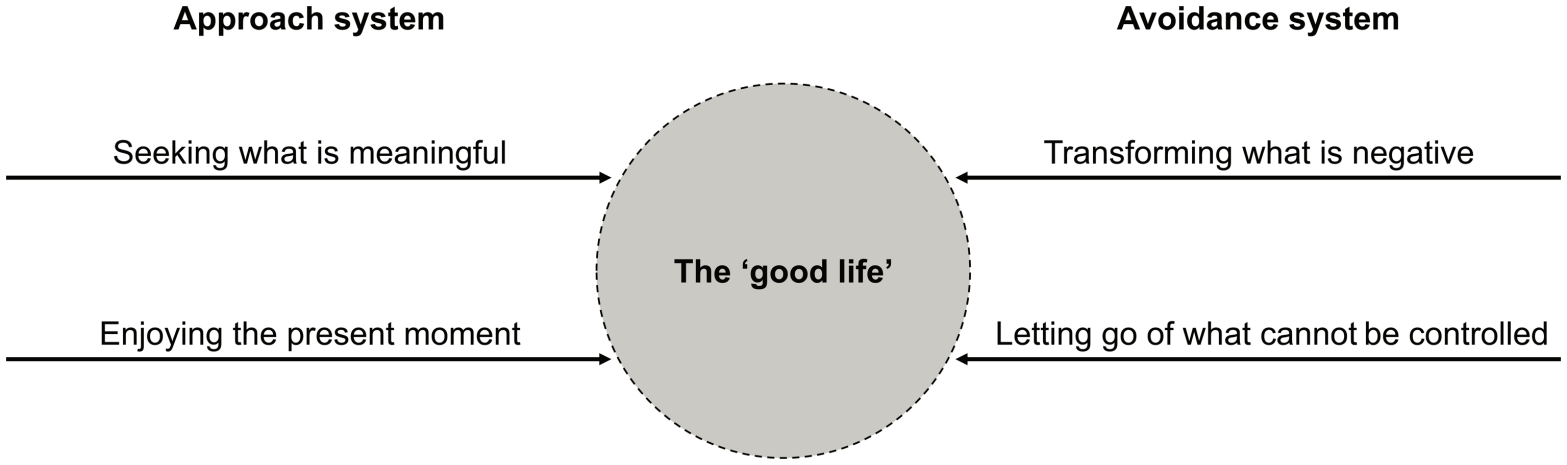
Visual illustration summarizing the dual-systems model reflecting the ‘good life.’

The book of *I-Ching* is the origin of various classic texts in ancient China. Yin-Yang is the root metaphor in the cultural system of I-Ching (Hwang, 2001), and it may be used to denote many opposite but interconnected forces in the universe (e.g., feminine-masculine, moon-sun, day-night; see Figure 1). However, Yin and Yang are not two mutually exclusive concepts. Rather, each contains a component of the other. The principle of Yin-Yang dialectical interactions has been translated into the dual-systems model (Wong, 2011, 2012). According to this model, the approach and avoidance systems co-exist and operate in an interdependent and complementary manner (see Figure 2 for a succinct illustration). This is somewhat analogous to the complementariness of the autonomic nervous system divisions (i.e., sympathetic vs. parasympathetic nervous system) or opponent processes involved in regulating attention (i.e., default vs. task-focused mode). The approach system represents appetitive behaviors, such as goal strivings, goal attainment, and positive emotions. The avoidance system represents defensive mechanisms against noxious stimuli, threats, dangers, and negative emotions. Depending on the context, balanced interaction between the two systems is thought to result in more adaptive success and vitality than focusing exclusively on either approach or avoidance. When a person is not actively engaged in approach or avoidance, the default or neutral stage is modulated by the awareness regulation system, which is best maintained by mindful awareness.

The integration of Yin-Yang dialectics to the interplay between approach and avoidance systems can enrich both academic and professional branches of psychology. For example, much of existing research in positive psychology tends to emphasize actions aimed at enhancing wellbeing, but the dual-systems perspective suggests that actions focused on transforming or transcending suffering are equally important for cultivating more durable wellbeing (Ho et al., 2022; Wong et al., 2022). Similarly, psychotherapy might be especially effective when client change centers not only on short-term symptom reduction, but also on assisting clients to discover and pursue a preferred meaningful future (Wong, 2010, 2016; Cowden et al., 2021b, 2022). Hence, the dialectics of the approach and avoidance systems could contribute in important ways to improving our understanding of the human condition, identifying potential pathways to promote human wellbeing, and informing clinical practice.

## The power of Wu-Wei

The concept of Wu-Wei has roots in Daoist culture, and can be roughly translated into ‘non-doing’ or ‘actionless action’ (Taylor, 1978). According to Daoism, the most important idea is that Dao (‘the Way’) imitates or follows the way of Nature. Therefore, Wu-Wei can be characterized as “acting effortlessly and spontaneously in perfect harmony with a normative standard” (Slingerland, 2000, p. 296). We are often faced with complicated situations in which the moral boundaries are blurred, or we get stuck on the horn of a dilemma without a clear sense of what to do. Rather than trying to force or act out of desire for a particular outcome, the most adaptive approach might be to pause or take a rest period—adopting Wu-Wei—before deciding how to respond (Wong, 2012). In this way, Wu-Wei involves mindfully observing the situation, considering all available options, and waiting patiently to make the right move.

A growing number of studies have documented evidence demonstrating the power of Wu-Wei in different situations. Xing and Sims (2012) found that senior Chinese bankers had a reflexive awareness of Wu-Wei that shaped the way they went about their work and interacted with other colleagues, including exercising flow (e.g., focusing on the work rather than the result) and self-protection (e.g., stopping at the right moment). Similarly, Roberts and Ertubey (2021) recently demonstrated the relevance of Wu-Wei to Western contexts. Their qualitative study of United Kingdom runners revealed several themes that reflected the role of Wu-Wei in supporting wellbeing, including approaching suffering with acceptance, gratitude, and an opportunity to grow, remaining fully focused on the present, and approaching life from a broader perspective. These findings suggest that practicing Wu-Wei can help people avoid unnecessary struggles and transcend negative experiences in ways that cultivate more enduring wellbeing. The non-duality of Wu-Wei can also have important broader implications for understanding and fostering deeper forms of happiness (e.g., mature happiness) that are less widely acknowledged or promoted in mainstream psychology.

## The doctrine of Zhong Yong

The doctrine of Zhong Yong is a central part of the cultural system of Confucianism (Li, 2008). A summary and second order interpretation of all Confucius’ teachings can be found in Chapter 20 of *Zhong Yong*, one of four classic books of Confucianism. The word Zhong literally means ‘center’ or ‘middle,’ and practically involves acting in a non-biased way. Yong refers to ‘usefulness’ or ‘constant.’ Together, Zhong Yong can be translated into ‘the mean as a constructive principle’ or ‘consistently practicing the middle-road in daily life’ (Suh, 2020; Gao et al., 2022). The Confucian doctrine of Zhong Yong shares some parallels with Aristotle’s doctrine of the Mean (e.g., both advocate that one can have too much or too little of something), but the former refers to the mentality of how to live a life of harmony whereas the latter is oriented principally toward transcendent virtue (Xia, 2020).

Following the path of Zhong Yong does not entail mechanically or quantitatively finding an equal distance between opposites, but instead is a qualitative state of equilibrium and harmony with oneself, the world around us, and the transcendental realm. For this reason, the doctrine of Zhong Yong is somewhat different from the optimality principle, which is essentially concerned with quantitatively maximizing benefits (Suh, 2020). The golden Mean emphasized by the doctrine of Zhong Yong is a harmonious state of balance between internal and external factors. It is also the fundamental criterion for achieving balanced harmony (Li and Cui, 2022), which is based on the ultimate balance of internal harmony (i.e., a harmonious state within one’s mind and body) and external harmony (i.e., harmonious relationships with others, with society, and with nature). On this view, the ideal life is to live in peace within oneself and in harmony with family members, neighbors, and nature.

When adversity and hardship arise, it is both unrealistic and difficult to maintain a positive emotional state because being in a happy mood while also experiencing anxiety about resolving a very stressful situation is physiologically infeasible. In such circumstances, the best we can hope for is to remain calm and maintain a delicate balance between anxiety and positive emotions, which means inner peace. This can be achieved by nurturing inner harmony with oneself and with others, gratitude, and contentment. Therefore, according to Asian psychology, optimal balanced harmony between self and others and between opposites in each situation can lead to mature happiness that is based on inner peace, harmony, and contentment (Wong and Bowers, 2019; Carreno et al., 2021).

The implication of Zhong Yong is that wellbeing for both the East and the West depends on achieving balanced harmony between internal and external harmony. In recognition of this, recent literature points to balance and harmony as the ‘golden thread’ that runs through all aspects of life (Lomas, 2021). Perhaps more importantly, large-scale global research through the Global Wellbeing Initiative (a partnership between Gallup and the Wellbeing for Planet Earth Foundation) is underway to explore experiences of balance and harmony around the world. This collaboration could play an important role in reshaping the WEIRD-centric database of existing research on wellbeing and catalyzing a more well-rounded understanding of human wellbeing, although further work will be needed to enrich the evidence that emerges from this effort with research that provides greater depth on balance, harmony, and other concepts that are particularly prominent in non-Western theologies and philosophies.

## Some strategies to enrich mainstream psychology

Everything that people do is shaped and colored by culture. Therefore, we can only develop a comprehensive understanding of human nature by studying more diverse participants and cultural contexts. To support further change toward an inclusive and representative global psychology, we propose some strategies (in no particular order) that may be useful to researchers who are interested in further transforming the WEIRD-centricity of mainstream psychology.

First, researchers are encouraged to adopt and refine integrative theories and conceptual models that attempt to address the complexities of the human condition and can support the needs of humanity. One possible prototype for a more global psychology is the multicultural existential positive psychology paradigm. On the surface, existential positive psychology is a synthesis of positive psychology and existentialism. At a deeper level, existential positive psychology is an integration of ancient Chinese positive psychology rooted in Taoism, Confucianism, and Buddhism, existential universals of ultimate concerns and transcendental values, and Western clinical and positive psychology. At its core, existential positive psychology is concerned with how to find harmony, contentment, and tranquility in the midst of constant change, chaos, and suffering by becoming attuned with oneself, humanity, and God or Tao (Chen, 2006; Hwang, 2012; Wong et al., 2021b). Accumulating literature suggests that existential positive psychology has heuristic value in advancing the science and practice of psychology (e.g., Wong, 2019; Arslan and Wong, 2021; Van Tongeren and Showalter Van Tongeren, 2021), and it has potential to make a substantive contribution to the next frontier of theory, research, and practice aimed at supporting the psychological needs of humanity.

Second, psychology ought to further develop, employ, and fine-tune multidimensional measures that address the non-dualistic and dialectical dynamics of human experience. For example, several measures have drawn on Yin-Yang dialectical interactions between positive and negative to assess phenomena of interest in a two-dimensional space (e.g., Death Attitude Profile-Revised; Wong et al., 1994) or the co-existence of conflicting emotions and experiences (Life Attitudes Scale-Brief; Leung et al., 2021). In principle, such measures are an attempt to align quantitative assessment of psychological phenomena more closely with the complex nature of human psychological experience. To the extent that assessment is successful at achieving this, it is possible that psychological science might evolve more rapidly.

Third, reforming the WEIRD-centricity of mainstream psychology will require more culturally responsive research that strikes a balance between upholding rigorous standards for causal inference and acquiring rich insights that support a more granulated interpretation of psychological phenomena within a particular culture and context. This could be accomplished (in part) through carefully orchestrated mixed methods research that draws on the best of robust, systematized quantitative approaches and creative, flexible qualitative techniques. For example, well-designed longitudinal studies are necessary for estimating causal effects with observational data, whereas qualitative approaches provide valuable opportunities to acquire a deeper understanding of the psychological processes and contextual factors that shape outcomes. Consistently applying this combination of research methodologies together may be indispensable for capturing the breadth and depth needed to fully address complex themes that are gaining popularity, such as the notion of transcending suffering as a foundation for flourishing (Wong et al., 2022; Wong and Tweed, 2022).

Fourth, further efforts must be dedicated toward developing, testing, and refining culturally responsive interventions and treatments informed by integrative models that synthesize disparate theoretical orientations (Wong, 2010; Cowden et al., 2023). Although psychotherapeutic treatments along these lines are essential, it will also be important to formulate culturally sensitive and contextually appropriate evidence-based interventions that have the potential for widespread dissemination at nominal cost (e.g., self-guided psychoeducational approaches; Wong et al., 2022). Successful prototypes from Western psychology (e.g., do-it-yourself REACH Forgiveness workbooks; Worthington, 2020) could provide useful templates for designing such scalable interventions. Although these kinds of interventions would not be an alternative to psychotherapy, they could be complementary to clinical treatment and also serve as valuable resources for non-treatment-seeking individuals who might benefit from additional supports.

The abovementioned strategies will need to be supported by changes in how research in psychology is typically conducted, funded, and evaluated. A truly global psychology needs programmatic lines of local, regional, and international research involving both WEIRD and non-WEIRD scholars and practitioners. Local scholars in non-WEIRD contexts might consider forming collaborative partnerships with well-established international researchers who could provide expertise in developing and implementing systematic programs of research (Cowden et al., 2023). International projects must include local researchers who reside in and have knowledge of the contexts under study, and it would be highly preferable to have psychologists from non-WEIRD countries leading the research teams for such projects (Wong et al., 2021a).

Arnett (2008) proposed several policies to improve the internationalization of psychological science, some of which aimed to alter traditional journal practices (e.g., encouraging prominent APA journals to include non-Western scholars as associate and consulting editors) and funding priorities (e.g., calling for funding agencies to create programs that fund international research). Although some progress has been made toward adopting the policies that were outlined, changes have mostly been incremental and quite limited. For example, Thalmayer et al. (2021) reported that inclusion of international scholars on editorial boards of APA journals remained relatively unchanged after approximately 10 years since Arnett’s (2008) policy proposals were made. We are optimistic about the changes that could occur if commitments were made to prioritize guidelines and propositions put forward by Arnett (2008) and others (e.g., Nielsen et al., 2017; Hendriks et al., 2019; Cheon et al., 2020), but comprehensive and sustained transformation seems unlikely unless all stakeholders with an interest in the discipline of psychology (e.g., researchers, practitioners, journal editors, journal reviewers, funding agencies) have a role in that process. For example, including non-Western associate and consulting editors on the editorial boards of high-impact journals might only have a partial impact on reducing bias toward concepts and findings from WEIRD researchers if journals do not also take steps to recruit and maintain a database of non-Western reviewers who can support a more balanced peer-review process. Similarly, funding agencies should consider instituting policies and practices that could meaningfully contribute to a less WEIRD database of evidence and promote longer-term changes in research that help to address concerns about the generalizability of psychological science, such as requiring a proportion of funded research projects to meet criteria that align with transforming the current WEIRDness of mainstream psychology. Therefore, a multilayered, systems-level approach might be needed to accomplish the scale of transformation that will lead to a global psychology.

## Conclusion

In recent years, there has been a clear shift away from a WEIRD-centric psychology to a more inclusive global psychology on several fronts, including an epistemological revolution to replace positivism with culture-inclusive psychological theories (Hwang, 2019) and a movement toward balancing self or ego-based Western psychotherapies with the self-enlightenment cultivation process that is based on Confucian, Buddhist, and Taoist teachings (Shiah, 2020). Complementing these efforts, this paper drew on principles from Asian psychology to highlight the potential for mainstream psychology to be reshaped into a scientific discipline that generates evidence which can be generalized more broadly beyond WEIRD societies. Although we have re-signaled the importance of ramping up efforts to develop a more inclusive global psychology, achieving such change will likely require collective commitment from psychologists in all parts of the world.

## Author contributions

PW and RC contributed equally to the article and approved the submitted version.

## Conflict of interest

The authors declare that the research was conducted in the absence of any commercial or financial relationships that could be construed as a potential conflict of interest.

## Publisher’s note

All claims expressed in this article are solely those of the authors and do not necessarily represent those of their affiliated organizations, or those of the publisher, the editors and the reviewers. Any product that may be evaluated in this article, or claim that may be made by its manufacturer, is not guaranteed or endorsed by the publisher.

## References

[ref1] Arnett J. J. (2008). The neglected 95%: why American psychology needs to become less American. Am. Psychol. 63, 602–614. doi: 10.1037/0003-066X.63.7.602, PMID: 18855491

[ref2] Arslan G. Wong P. T. P. (2021). Measuring personal and social responsibility: an existential positive psychology approach. J. Happiness Health 2, 1–11. doi: 10.47602/johah.v2i1.5

[ref3] Carreno D. F. Eisenbeck N. Pérez-Escobar J. A. García-Montes J. M. (2021). Inner harmony as an essential facet of well-being: a multinational study during the COVID-19 pandemic. Front. Psychol. 12:648280. doi: 10.3389/fpsyg.2021.648280, PMID: 33841286 PMC8034265

[ref4] Chen Y. H. (2006). “Coping with suffering: the Buddhist perspective” in Handbook of multicultural perspectives on stress and coping. eds. Wong P. T. P. Wong L. C. J. (New York, NY: Springer), 73–89

[ref5] Cheon B. K. Melani I. Hong Y.-Y. (2020). How USA-centric is psychology? an archival study of implicit assumptions of generalizability of findings to human nature based on origins of study samples. Soc. Psychol. Personal. Sci. 11, 928–937. doi: 10.1177/1948550620927269

[ref6] Cowden R. G. Counted V. Ho M. Y. (2023). “Positive psychology and religion/spirituality across cultures in Africa, Asia, and Oceania” in Handbook of positive psychology, religion, and spirituality. eds. Davis E. B. Worthington E. L. Jr. Schnitker S. A. (Cham, Switzerland: Springer), 243–259.

[ref7] Cowden R. G. Davis E. B. Counted V. Chen Y. Rueger S. Y. VanderWeele T. J. (2021b). Suffering, mental health, and psychological well-being during the COVID-19 pandemic: a longitudinal study of U.S. adults with chronic health conditions. Wellbeing Space Soc. 2:100048. doi: 10.1016/j.wss.2021.100048, PMID: 34746895 PMC8562865

[ref8] Cowden R. G. Mascret N. Duckett T. R. (2021a). A person-centered approach to achievement goal orientations in competitive tennis players: associations with motivation and mental toughness. J. Sport Health Sci. 10, 73–81. doi: 10.1016/j.jshs.2018.10.001, PMID: 33518017 PMC7856560

[ref9] Cowden R. G. Wȩziak-Białowolska D. McNeely E. VanderWeele T. J. (2022). Are depression and suffering distinct? an empirical analysis. Front. Psychol. 13:970466. doi: 10.3389/fpsyg.2022.970466, PMID: 36186371 PMC9518749

[ref10] Gao R. Huang S. Yao Y. Liu X. Zhou Y. Zhang S. (2022). Understanding Zhongyong using a Zhongyong approach: re-examining the non-linear relationship between creativity and the Confucian doctrine of the mean. Front. Psychol. 13:903411. doi: 10.3389/fpsyg.2022.903411, PMID: 35783697 PMC9240665

[ref11] Hendriks T. Warren M. A. Schotanus-Dijkstra M. Hassankhan A. Graafsma T. Bohlmeijer E. (2019). How WEIRD are positive psychology interventions? a bibliometric analysis of randomized controlled trials on the science of well-being. J. Posit. Psychol. 14, 489–501. doi: 10.1080/17439760.2018.1484941

[ref12] Henrich J. Heine S. Norenzayan A. (2010). Most people are not WEIRD. Nature 466:29. doi: 10.1038/466029a 20595995

[ref13] Ho S. Cook K. V. Chen Z. J. Kurniati N. M. T. Suwartono C. Widyarini N. (2022). Suffering, psychological distress, and well-being in Indonesia: a prospective cohort study. Stress Health. doi: 10.1002/smi.3139 [Epub ahead of print]. PMC10078741 35244330

[ref14] Hwang K.-K. (2001). The deep structure of Confucianism: a social psychological approach. Asian Philo. 11, 179–204. doi: 10.1080/09552360120116928

[ref15] Hwang K.-K. (2012). Foundations of Chinese psychology: Confucian social relations. New York, NY: Springer.

[ref16] Hwang K.-K. (2015). Culture-inclusive theories of self and social interaction: the approach of multiple philosophical paradigms. J. Theory Soc. Behav. 45, 40–63. doi: 10.1111/jtsb.12050

[ref17] Hwang K.-K. (2019). Culture-inclusive theories: An epistemological strategy. Cambridge, UK: Cambridge University Press.

[ref19] Leung M. M. Arslan G. Wong P. T. P. (2021). Tragic optimism as a buffer against COVID-19 suffering and the psychometric properties of a brief version of the Life Attitudes Scale. Front. Psychol. 12:646843. doi: 10.3389/fpsyg.2021.646843, PMID: 34552523 PMC8450366

[ref20] Li C. (2008). The philosophy of harmony in classical Confucianism. Philos Compass 3, 423–435. doi: 10.1111/j.1747-9991.2008.00141.x

[ref21] Li Y. Cui H. (2022). On the value of the Chinese pre-Qin Confucian thought of "harmony" for modern public mental health. Front. Psychol. 13:870828. doi: 10.3389/fpsyg.2022.870828, PMID: 35719596 PMC9203267

[ref22] Lomas T. (2018). Experiential cartography and the significance of “untranslatable” words. Theory Psychol. 28, 476–495. doi: 10.1177/0959354318772914

[ref23] Lomas T. (2021). Life balance and harmony: wellbeing’s golden thread. Int. J. Wellbeing 11, 50–68. doi: 10.5502/ijw.v11i1.1477

[ref25] Nielsen M. Haun D. Kärtner J. Legare C. H. (2017). The persistent sampling bias in developmental psychology: a call to action. J. Exp. Child Psychol. 162, 31–38. doi: 10.1016/j.jecp.2017.04.017, PMID: 28575664 PMC10675994

[ref26] Roberts W. Ertubey C. (2021). Flow the wu-wei way: a thematic analysis of charity runners’ experience of wu-wei in enhancing wellbeing and flourishing. Int. J. Wellbeing 12, 132–154. doi: 10.5502/ijw.v12i4.2129

[ref27] Shiah Y.-J. (Ed.) (2020). Foundations of Chinese psychotherapies: Towards self-enlightenment. Cham, Switzerland: Springer.

[ref28] Slingerland E. (2000). Effortless action: the Chinese spiritual ideal of wu-wei. J. Am. Acad. Relig. 68, 293–328. doi: 10.1093/jaarel/68.2.293

[ref29] Suh J. (2020). The Confucian doctrine of the mean, the optimality principle, and social harmony. Soc. Econ. 42, 59–73. doi: 10.1556/204.2020.00004

[ref30] Taylor E. (1978). “Asian interpretations: transcending the stream of consciousness” in The stream of consciousness: Scientific investigations into the flow of human experience. eds. Pope K. S. Singer J. L. (New York, NY: Springer), 31–54.

[ref31] Thalmayer A. G. Toscanelli C. Arnett J. J. (2021). The neglected 95% revisited: is American psychology becoming less American? Am. Psychol. 76, 116–129. doi: 10.1037/amp0000622, PMID: 32271027

[ref33] Van Tongeren D. R. Showalter Van Tongeren S. A. (2021). Finding meaning amidst COVID-19: an existential positive psychology model of suffering. Front. Psychol. 12:641747. doi: 10.3389/fpsyg.2021.641747, PMID: 33776866 PMC7987806

[ref34] Wang Z. Shakeshaft N. Schofield K. Malanchini M. (2018). Anxiety is not enough to drive me away: a latent profile analysis on math anxiety and math motivation. PLoS ONE 13:e0192072. doi: 10.1371/journal.pone.0192072, PMID: 29444137 PMC5812593

[ref35] Wong P. T. P. (2010). Meaning therapy: an integrative and positive existential psychotherapy. J. Contemp. Psychother. 40, 85–93. doi: 10.1007/s10879-009-9132-6

[ref36] Wong P. T. P. (2011). Positive psychology 2.0: towards a balanced interactive model of the good life. Can. Psychol. 52, 69–81. doi: 10.1037/a0022511

[ref37] Wong P. T. P. (2012). “Toward a dual-systems model of what makes life worth living” in The human quest for meaning: Theories, research, and applications. ed. Wong P. T. P.. 2nd ed (New York, NY: Routledge), 3–22.

[ref38] Wong P. T. P. (2016). Self-transcendence: a paradoxical way to become your best. Int. J. Existential Positive Psychol. 6.

[ref39] Wong P. T. P. (2019). Second wave positive psychology’s (PP 2.0) contribution to counselling psychology. Couns. Psychol. Q. 32, 275–284. doi: 10.1080/09515070.2019.1671320

[ref40] Wong P. T. P. Arslan G. Bowers V. L. Peacock E. J. Kjell O. N. E. Ivtzan I. (2021a). Self-transcendence as a buffer against COVID-19 suffering: the development and validation of the Self-Transcendence Measure-B. Front. Psychol. 12:648549. doi: 10.3389/fpsyg.2021.648549, PMID: 34690853 PMC8527188

[ref41] Wong P. T. P. Bowers V. (2019). “Mature happiness and global wellbeing in difficult times” in Scientific concepts behind happiness, kindness, and empathy in contemporary society. ed. Silton N. R. (Hershey, PA: IGI Global), 112–134.

[ref42] Wong P. T. P. Cowden R. G. Mayer C.-H. Bowers V. L. (2022). “Shifting the paradigm of positive psychology: toward an existential positive psychology of wellbeing” in Broadening the scope of wellbeing science: Multidisciplinary and interdisciplinary perspectives on human flourishing and wellbeing. eds. Kemp A. H. Edwards D. J. (Cham, Switzerland: Palgrave Macmillan), 13–27.

[ref43] Wong P. T. P. Mayer C.-H. Arslan G. (2021b). Editorial: COVID-19 and existential positive psychology (PP 2.0): the new science of self-transcendence. Front. Psychol. 12:800308. doi: 10.3389/fpsyg.2021.800308, PMID: 34956025 PMC8699172

[ref44] Wong P. T. P. Reker G. T. Gesser G. (1994). “Death attitude profile–revised: a multidimensional measure of attitudes toward death” in Death anxiety handbook: Research instrumentation and application. ed. Neimeyer R. A. (Cham, Switzerland: Taylor and Francis), 121–148.

[ref45] Wong P. T. P. Sproule C. F. (1984). “Attributional analysis of locus of control and the Trent Attribution Profile (TAP)” in Research with the locus of control construct, Vol. 3: Limitations and extensions. ed. Lefcourt H. M. (Washington, DC: Academic Press), 309–360.

[ref46] Wong P. T. P. Tweed R. (2022). “Region of upper North America (United States and Canada)” in The international handbook of positive psychology: A global perspective on the science of positive human existence. eds. Chang E. C. Downey C. Yang H. Zettler I. Muyan-Yılık M. (Cham, Switzerland: Springer), 17–47.

[ref47] Worthington E. L. Jr. (2020). “An update of the REACH Forgiveness model: psychoeducation in groups, do-it-yourself formats, couple enrichment, religious congregations, and as an adjunct to psychotherapy” in Handbook of forgiveness. *2nd* edn. Worthington E. L. Jr. Wade N. G. (New York, NY: Routledge), 277–287.

[ref50] Xia F. A. (2020). Comparative study of Aristotle’s doctrine of the Mean and Confucius’ doctrine of Zhong Yong. Int. Commun. Chin. Cult. 7, 349–377. doi: 10.1007/s40636-020-00194-x

[ref48] Xing Y. Sims D. (2012). Leadership, Daoist Wu Wei and reflexivity: flow, self-protection and excuse in Chinese bank managers’ leadership practice. Manag. Learn. 43, 97–112. doi: 10.1177/1350507611409659

